# Clustering of Urinary Biomarkers to Identify Interstitial Cystitis Subtypes and Different Clinical Characteristics and Treatment Outcomes

**DOI:** 10.3390/biomedicines13020369

**Published:** 2025-02-05

**Authors:** Jing-Hui Tian, Chung-You Tsai, Wan-Ru Yu, Yuan-Hong Jiang, Jia-Fong Jhang, Hann-Chorng Kuo

**Affiliations:** 1 Department of Urology, Hualien Tzu Chi Hospital, Buddhist Tzu Chi Medical Foundation, and Tzu Chi University, Hualien 970374, Taiwan; luckysweet999@gmail.com (J.-H.T.); wanzu666@gmail.com (W.-R.Y.); redeemerhd@gmail.com (Y.-H.J.); alur1984@hotmail.com (J.-F.J.); 2 Divisions of Urology, Department of Surgery, Far Eastern Memorial Hospital, New Taipei 220216, Taiwan; pgtsai@gmail.com; 3 Department of Electrical Engineering, Yuan Ze University, Taoyuan 320315, Taiwan

**Keywords:** bladder pain syndrome, urinary biomarker, cystitis, urine cytokines, women

## Abstract

**Purpose:** Interstitial cystitis/bladder pain syndrome (IC/BPS) is mysterious and difficult to diagnose without cystoscopic hydrodistention. This study aimed to explore non-invasive and highly reliable urine biomarkers to identify Hunner’s IC (HIC) and different non-Hunner’s IC (NHIC) subtypes. **Methods:** In total, 422 women with and without clinically diagnosed IC/BPS (n = 376 and 46, respectively) were retrospectively enrolled. Patients were diagnosed with HIC or NHIC by cystoscopic hydrodistention under anesthesia. Then, the maximal bladder capacity (MBC) and glomerulation grade were determined. Thirteen urine inflammatory cytokines, chemokines, and oxidative stress biomarkers based on the previously reported predictors of IC/BPS were assayed using commercial microsphere kits. The dataset was randomly divided into training (70%) and test (30%) sets for model construction and validation using logistic regression and stepwise variable selection techniques. To construct the predictive models, univariate analysis was performed to evaluate the discriminative power of each urinary biomarker, measured by the area under the curve (AUC). Biomarkers with AUC values < 0.6 were excluded from further modeling. Multivariate logistic regression was then employed, with variables selected through stepwise forward selection based on log-likelihood criteria. For dichotomization, cutoff values were determined using quartile ranges from the control group. The final model’s performance was assessed using AUC, accuracy, sensitivity, and specificity in both training and test sets. **Results:** By setting the screening criterion to AUC ≥ 0.60, the potential urinary biomarkers for identifying IC/BPS cases were eotaxin, monocyte chemoattractant protein-1, tumor necrosis factor-alpha (TNF-α), 8-hydroxy-2′-deoxyguanosine (8-OHdG), and 8-isoprostane. Those for identifying HIC from the IC/BPS cohort were interleukin (IL)-6, IL-8, interferon γ-inducible protein 10 (IP-10), and regulated on activation, normal T-cell expressed and secreted (RANTES). A diagnostic algorithm using a cluster of urinary biomarkers included TNF-α ≥ 0.95 pg/mL or 8-OHDG ≥ 22.34 pg/mL and 8-isoprastane ≥ 22.34 pg/mL for identifying IC/BPS from the overall cohort; for identifying HIC from the IC/BPS cohort, the urinary IP-10 ≥ 3.74 pg/mL or IP-10 ≥ 19.94 pg/mL was added. **Conclusions:** Using a cluster of urinary biomarkers such as TNF-α or 8-OHdG and 8-isoprostane can identify IC/BPS from a study cohort, and adding the urinary IP-10 can distinguish HIC from IC/BPS cases.

## 1. Introduction

Interstitial cystitis/bladder pain syndrome (IC/BPS) is a condition characterized by the clinical symptoms of bladder pain or discomfort and bladder irritative symptoms such as urgency and frequency [1]. Although IC/BPS is not life threatening, it greatly impairs patients’ quality of life, with no durable and effective treatment [2]. IC/BPS has different subtypes, including ulcerative IC and nonulcerative IC, which have distinct pathophysiological and clinical presentations [3]. The actual pathophysiology of IC/BPS is still unclear; however, according to current evidence, chronic inflammation, sensory hyperinnervation, urothelial dysregulation, urothelial barrier deficits, and central nervous system sensitization might be involved in IC/BPS pathogenesis at different levels [4]. Persistent inflammation enhances the apoptotic pathway, causing decreased proliferation and the maturation of the urothelial cells, and finally, apical cell deficits and barrier function impairment [5].

Depending on the level of pathogenesis, the IC/BPS bladders might present with different cystoscopic characteristics, including IC/BPS bladders with Hunner’s lesion or Hunner’s IC (HIC) and non-Hunner’s IC (NHIC) [3]. NHIC can also present with a different maximal bladder capacity (MBC) and glomerulation grade after cystoscopic hydrodistention under anesthesia [6]. However, lower urinary tract symptoms alone cannot provide an accurate diagnosis, and the differential diagnosis of various IC/BPS subtypes requires invasive cystoscopic hydrodistention and bladder biopsy [1]. Patients who are late to be diagnosed for IC/BPS may suffer from long-term bladder pain and a low quality of life. Searching for a diagnostic tool for IC/BPS is an unmet need.

The treatments of HIC and NHIC are distinct [2]. Because the bladder in HIC comprises dense inflammatory tissue, therefore, the fulguration or resection of the bladder wall with Hunner’s lesion is the first step and the therapeutic effect is expectable [7]. However, although numerous treatment guidelines have outlined various therapeutic options, there is no effective and durable treatment for all subtypes of NHIC [8]. Recently, novel treatments such as Botulinum toxin A, platelet-rich-plasma, and low-energy shock wave therapy have been reported to have satisfactory rates of only around 47.5 to 67.5% [9,10,11], and without considering patient phenotypes [6]. Thus, exploring the high reliability of biomarkers for predicting IC/BPS could help patients achieve a more satisfactory treatment outcome, which is the ultimate goal. Integrating urinary biomarkers into clinical practice offers a promising avenue for achieving these goals. If certain biomarkers can reliably reflect specific pathophysiological mechanisms in IC/BPS, they could inform personalized treatment strategies that address the unique needs of each patient.

Urinary inflammatory proteins, neurogenic proteins, and oxidative stress biomarkers are significantly elevated in patients with lower urinary tract dysfunctions (LUTDs) [12]. Urinary biomarker measurement could provide evidence for the differential diagnosis of LUTDs with similar clinical symptoms such as hypersensitive bladder, overactive bladder, detrusor overactivity, dysfunctional voiding, mixed urinary incontinence, bladder outlet obstruction, and IC/BPS [13]. Oxidative stress biomarkers also play a diagnostic role in discriminating IC/BPS from controls and in identifying HIC from NHIC cases [14].

Furthermore, both the serum and urinary biomarkers have been enthusiastically investigated to identify IC/BPS, to assess the treatment outcome, and to predict the prognosis of different intravesical therapies, including the antiproliferative factor, vascular endothelial growth factor, epidermal growth factor, and hypoxia-inducible factor [15,16,17]. These serum and urinary biomarkers might be sensitive enough to identify IC/BPS, but their specificity is not high; thus, they are not widely applied in the differential diagnosis of LUTDs clinically [18]. A single urinary biomarker might not accurately identify IC/BPS subtypes, but using a cluster of urinary biomarkers, we might identify IC/BPS from patients with a hypersensitive bladder, as well as providing treatment guidance and prognosis for IC/BPS [19]. In this study, we aimed to identify the clinical subtypes of IC/BPS and HIC by using a cluster of urinary biomarkers that had been previously reported to be sensitive in identifying patients with IC/BPS.

## 2. Materials and Methods

The diagnostic criteria for IC/BPS were based on the Asian IC guidelines and the exclusion of confusable diseases as shown in the ESSIC guidelines [8,20,21]. Our participating patients had been enrolled in previous clinical studies approved by the institutional review board and ethics committee of the hospital (approval no.: 113-151-B). All patients had participated in different clinical investigations and trials for IC/BPS treatment. Furthermore, they were informed about the study purpose before providing consent. However, the need for informed consent was waived in this retrospective study because urine samples were collected and approved for use in previous clinical trials.

### 2.1. Patient Characteristics

We retrospectively enrolled 422 women with clinically diagnosed IC/BPS (n = 376) and non-IC/BPS (n = 46). For the clarification of different IC/BPS subtypes, we named patients with Hunner’s lesion as the HIC group and those without Hunner’s lesion as the NHIC group. When the symptoms or urinary biomarkers were related to both the HIC and NHIC groups, the name “IC/BPS” is given [1,2]. All patients had undergone a videourodynamic study before admission to exclude those with LUTDs, such as detrusor overactivity, bladder outlet obstruction, dysfunctional voiding, and neurogenic bladder [13]. HIC diagnosis was made using office cystoscopy without anesthesia according to the characteristic cystoscopic findings of HIC [6,22]. For NHIC diagnosis, cystoscopic hydrodistention under general anesthesia was used. The cystoscopic hydrodistention was performed under an intravesical pressure of 80 cm H_2_O for 10 min. Post-hydrodistention characteristic cystoscopic findings such as petechia, glomerulation, splotch hemorrhage, and mucosal fissures were recorded [6]. The glomerulation grade was classified according to the Asian IC guidelines [7]. In total, 30 women with genuine stress urinary incontinence but without other storage or voiding dysfunctions and 16 women with hypersensitive bladder but without characteristic NHIC cystoscopic findings were included in the control group. Our detailed inclusion and exclusion criteria are similar to those in our previous study [13]. On the basis of the glomerulation grade and MBC, patients with NHIC were further classified into different clinical subgroups for comparison [6].

### 2.2. Assessment of the Urinary Biomarkers

The urinary biomarker assessments were performed according to our previous study [23]. Briefly, 50 mL of urine samples was collected from the patients and controls before cystoscopic hydrodistention. These urine samples were obtained via self-voiding upon full bladder. Those collected from patients with confirmed urinary tract infection were excluded. The included samples were placed immediately on ice before being transported to the laboratory. Next, they were centrifuged at 1800 rpm for 10 min at 4 °C. The supernatant was frozen at −80 °C for preservation. The frozen urine samples were centrifuged at 12,000 rpm for 15 min at 4 °C before further analyses and subsequent measurements.

We used commercial microspheres to assay inflammation-associated urinary cytokines and chemokines using the Milliplex^®^ Human cytokine/chemokine magnetic bead-based panel kit (Millipore, Darmstadt, Germany). Urinary cytokines and chemokines were considered important in IC/BPS diagnosis. Thus, we selected 10 targeted analytes: interleukin (IL)-8; C-X-C motif chemokine ligand 10 (CXCL10) (interferon γ (IFNγ)-inducible protein 10, IP-10); monocyte chemoattractant protein-1 (MCP-1); eotaxin; IL-6; macrophage inflammatory protein 1 beta (MIP-1β); regulated on activation, normal T-cell expressed and secreted (RANTES); tumor necrosis factor alpha (TNF-α) (catalog number: HCYTA-60K); brain-derived neurotrophic factor (BDNF) (catalog number: HNDG3MAG-36K); and prostaglandin E2 (PGE2) (Cayman Chemical Co., Ann Arbor, MI, USA, No. 514010). These analytes were then measured using the multiplex kit (catalog number: HCYTMAG-60K-PX30). The procedures for measuring urinary cytokine and chemokine levels were performed according to the manufacturer’s instructions and the method utilized in previous studies [23]. The median fluorescence intensity of each cytokine/chemokine target was recorded and analyzed to calculate the individual corresponding cytokine/chemokine concentration in the urinary samples.

Moreover, 8-hydroxy-2′-deoxyguanosine (8-OHdG), 8-isoprostane, and total antioxidant capacity (TAC) in the urine samples were quantified according to the manufacturer’s instructions (8-OHdG ELISA kit from Biovision, Waltham, MA, USA, K4160-100; 8-isoprostane ELIZA kit from Enzo, Farmingdale, NY, USA, DI-900-010; and TAC Assay Kit from Abcam, Cambridge, MA, USA, ab52635). The procedures used in the urine biomarker assay were based on our previous report [14]. We quantified 8-isoprostane in the samples according to the manufacturer’s instructions (8 isoprostane ELISA kit, Enzo). Urine 8-isoprostane level measurements were standardized according to urinary creatinine levels measured using a commercial kit (Enzo Life Sciences Inc., Farmingdale, NY, USA, ADI-907-030A). The median fluorescence intensities of the targets were analyzed to calculate the corresponding concentrations of the urinary biomarkers in the samples. The TACs in the samples were also quantified. Briefly, 100 μL of Cu^2+^ working solution and 100 μL of the sample were sequentially added to 96-well plates (panel kits), which were then incubated for 90 min at room temperature on a shaker protected from the light. Finally, the plates were evaluated on the microplate reader at 570 nm. Likewise, we analyzed the median fluorescence intensities of the target to calculate the corresponding TAC concentrations in the samples.

### 2.3. Model Construction and Statistical Analysis

The patient cohort, which was defined according to the aforementioned criteria as the gold standard, was divided into two groups: IC/BPS and controls. The IC/BPS group was further divided into two subgroups: HIC and NHIC. Two separate predictive models were developed: Model 1, which aimed to predict the IC/BPS status, and Model 2, which focused on differentiating between HIC and NHIC within the IC/BPS population. Both models were designed as binary classification tasks.

For model training and validation, the entire dataset was randomly split into training and test sets (70% and 30%, respectively). Log transformation was applied to biomarker concentrations during data preprocessing to correct the right-skewness distribution. Initially, predictors for both models were selected from 13 urinary biomarkers. Each biomarker’s discriminative ability was evaluated in a univariate analysis by assessing its area under the curve (AUC) for predicting the target outcome. Biomarkers with minimal or no discriminative ability (AUC < 0.6) were excluded from further modeling.

Both Models 1 and 2 were constructed using a multivariate logistic regression analysis with the training set. The predictor variables were selected through a stepwise forward selection process based on the log-likelihood criteria. The discriminative performance of each model was evaluated on the test set using the AUC, accuracy, sensitivity, and specificity as performance metrics. To dichotomize continuous predictors into categorical variables, we utilized the concentrations of urinary biomarkers from the control as a reference. Each predictor’s concentration in the control group was divided into quartiles according to expression levels, with Quartile 2 (Q2) and Quartile 3 (Q3) chosen as the cutoff points.

Continuous variables with a normal distribution are presented as the mean ± standard deviation, with the *t*-test used for group comparisons. For comparing categorical variables, we used the chi-square test or Fisher’s exact test, as appropriate. Logistic regression results are presented as odds ratios with 95% confidence intervals. All statistical tests were two-tailed, with a significance level of *p* ≤ 0.05. All statistical data were compared using SPSS version 27 and R version 4.x.

## 3. Results

This study enrolled 422 patients, comprising 376 with HIC (n = 42) or NHIC (n = 334) and 46 without IC/BPS (including 30 with normal and 16 with hypersensitive bladder) from February 2010 to December 2021. The mean age was 53.9 ± 13.2 years. All patients were randomly divided into a training set (70%, n = 297) and a test set (30%, n = 125). All demographic characteristics, clinical features, and urine cytokine variables did not significantly differ between the training and test sets (Table 1 and Table 2).

**Figure 1:**
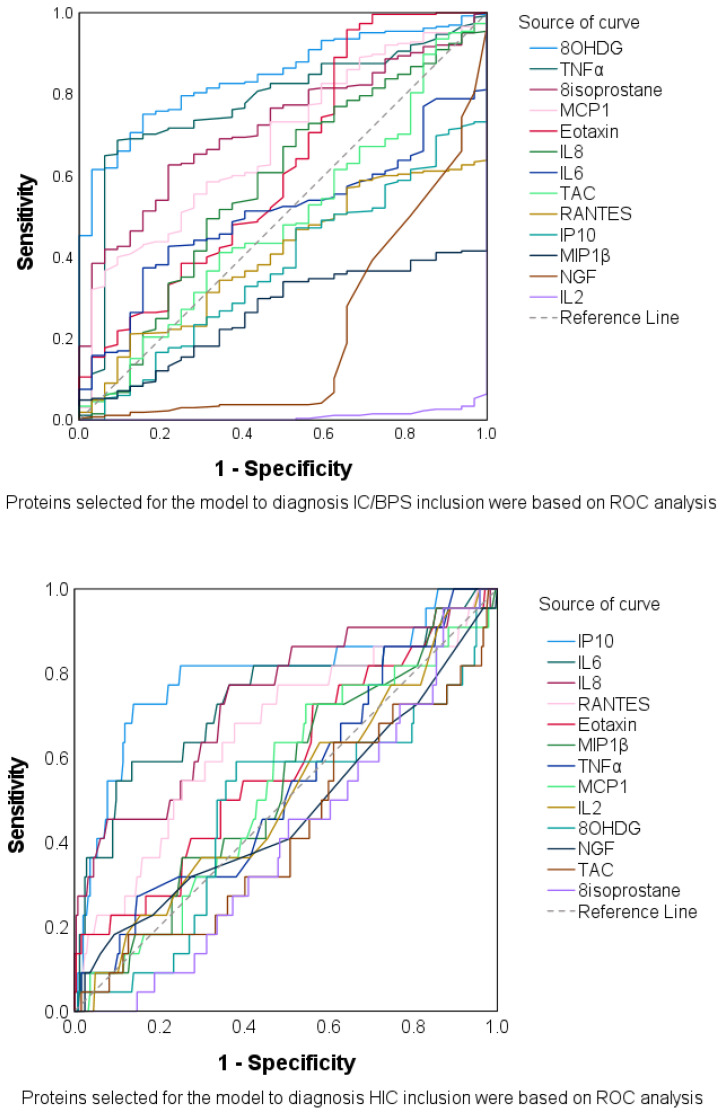
Receiver operating characteristic curves for all urinary biomarkers to identify interstitial cystitis/bladder pain syndrome (IC/BPS) from the total study cohort, and HIC from all patients with IC/BPS.

The AUC in all urinary biomarkers in the training set was investigated using the receiver operating characteristic (Figure 1). By setting the screening criterion to AUC ≥ 0.60, the urinary biomarkers eotaxin, MCP-1, TNF-α, 8-OHdG, and 8-isoproatane were found acceptable for identifying patients with IC/BPS. Meanwhile, IL-6, IL-8, IP-10, and RANTES were acceptable for identifying HIC from all patients with IC/BPS (Table 3).

To search for the optimal cutoff values of the urinary biomarkers to identify patients with IC/BPS and HIC, we calculated the urinary levels of individual biomarkers in the control group by quartile measurement and determined the urinary levels at CQ1, CQ2, and CQ3 (Table 4). Then, using the forward stepwise multivariate analysis, we established the models for identifying the IC/BPS and HIC cases.

On the basis of the forward stepwise multivariate analysis, patients with IC/BPS can be identified from the overall cohort (Model 1) through the following: TNF-α ≥ CQ2, 8-OHDG ≥ CQ2, or 8-isoprostane ≥ CQ2, or TNF-α ≥ CQ3, 8-OHdG ≥ CQ3, or 8-isoprostane ≥ CQ3. To identify patients with HIC from the IC/BPS group, we used IP-10 ≥ CQ2 or IP-10 ≥ CQ3 (Model 2) (Table 5).

By clustering the urinary biomarkers with AUC ≥ 0.60, a combination of TNF-α ≥ CQ3 or 8-OHdG ≥ CQ3 and 8-isoprostane ≥ CQ2 yielded a sensitivity of 76.2%, specificity of 75.0%, an accuracy of 76.1% in the training set (AUC = 0.76), and 78.4%, 71.4%, and 77.6% (AUC = 0.75), respectively, in the test set for identifying IC/BPS from the overall cohort. In the IC/BPS group, adding urinary IP-10 ≥ CQ3 yields sensitivities of 68.2% and 70.0%, specificities of 86.8% and 80.2%, and accuracies of 85.3% and 78.4% in the training set (AUC = 0.78) and in the test set (AUC = 0.75), respectively, for identifying patients with HIC (Table 6).

**Figure 2:**
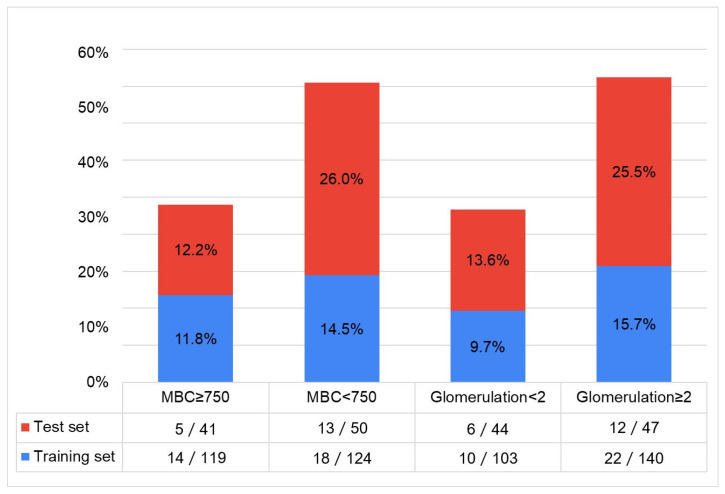
By clustering the urinary biomarkers of the combination of TNF-α ≥ CQ3 or 8-OHdG ≥ CQ3 and 8-isoprostane ≥ CQ2 in the training set and test set for identifying IC/BPS. Adding urinary IP-10 ≥ CQ3 to classify different bladder conditions involving MBC and the glomerulation grade in the NHIC group, the percentage was significantly higher in patients with MBC < 750 mL and glomerulation grade ≥ 2 than in those with MBC ≥ 750 mL or a glomerulation grade < 2.

When we added IP-10 ≥ CQ3 to classify different bladder conditions involving the MBC and glomerulation grade in the NHIC group, the percentage was significantly higher in patients with MBC < 750 mL and a glomerulation grade ≥ 2 than in those with MBC ≥ 750 mL or glomerulation grade < 2 (Figure 2). However, although an IP-10 ≥ CQ3 might indicate a higher grade of inflammation in patients with lower MBC and higher glomerulation grade, the sensitivity and specificity of identifying different combinations of MBC and glomerulation grades in NHIC were low, thereby likely unsuitable for identifying bladder condition in patients with NHIC.

**Figure 3:**
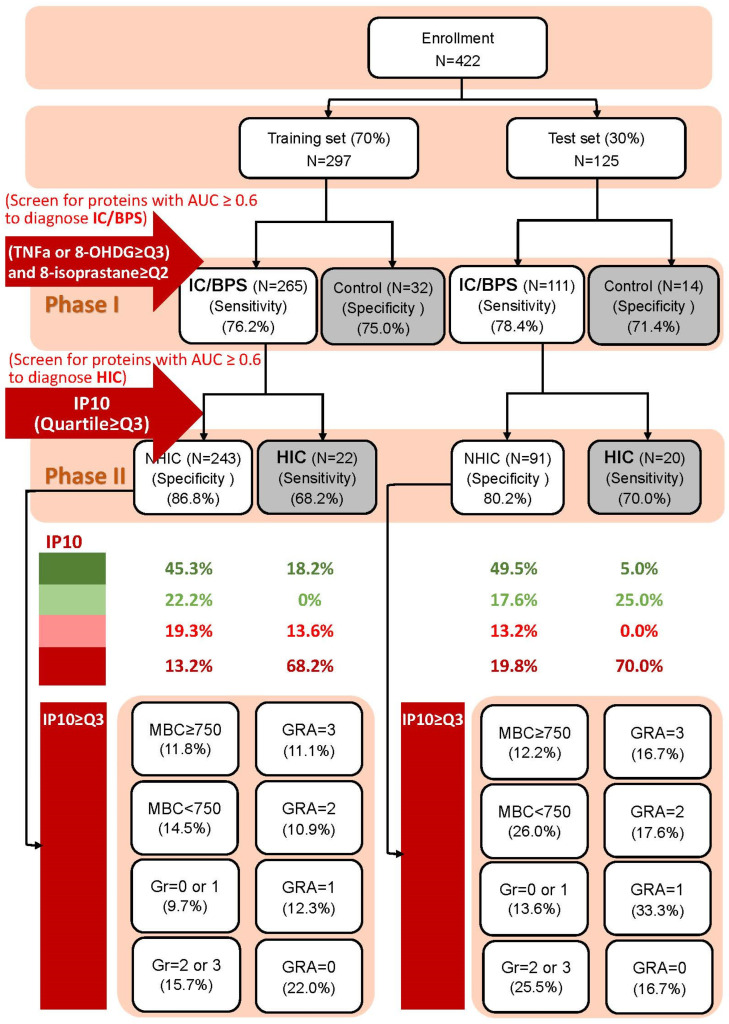
Diagnostic algorithm using a cluster of urinary biomarkers, namely TNF-α ≥ CQ3 or 8-OHDG ≥ CQ3, and 8-isoprastane ≥ CQ2, to identify interstitial cystitis/bladder pain syndrome (IC/BPS) and non-IC/BPS, and the addition of the urinary biomarker IP-10 ≥ CQ3 to identify Hunner’s IC (HIC) from all patients with IC/BPS.

According to the results shown above, a diagnostic algorithm using a cluster of urinary biomarkers including TNF-α ≥ CQ3 or 8-OHDG ≥ CQ3 and 8-isoprastane ≥ CQ2 could identify patients with IC/BPS from the overall cohort, and adding the urinary IP-10 ≥ CQ2 or IP-10 ≥ CQ3 could detect HIC from the IC/BPS population (Figure 3).

## 4. Discussion

This study revealed that using a cluster of urinary biomarkers such as TNF-α ≥ CQ3 (0.95 pg/mL) or 8-OHdG ≥ CQ3 (22.34 pg/mL) and 8-isoprostane ≥ CQ2 (13.35 pg/mL) can identify patients with IC/BPS from the study cohort and that adding the urinary IP-10 ≥ CQ3 (19.94 pg/mL) can identify HIC from the IC/BPS cohort. Thus, these urinary biomarkers can be used to screen patients who have characteristic symptoms without the need to undergo invasive diagnostic procedures.

IC/BPS can be divided into different subtypes, namely HIC and NHIC, which have distinct pathophysiological and clinical presentations. Urothelial dysfunction, chronic submucosal inflammation, and central sensitization are the most common IC/BPS pathogeneses [24]. The underlying pathophysiology is related to chronic inflammatory cell infiltration and inflammatory protein release [25,26]. Although IC/BPS has no durable therapy, the treatment strategy for HIC and NHIC is distinct. Even in patients with NHIC, the cystoscopic characteristic findings after hydrodistention are variable, likely resulting from different underlying pathophysiologies and different treatment modalities [6]. Noninvasive markers are needed for the differential diagnoses of the IC/BPS subtypes, especially HIC and NHIC, and different MBC and glomerulation grades in NHIC, as well as for differentiating IC/BPS from bladder sensory disorders, such as hypersensitive bladder syndrome or overactive bladder [23,27]. Analyzing multiple urinary proteins and serum cytokines might provide a diagnostic basis for IC/BPS [28].

The recent development of Milliplex for measuring urinary biomarkers to assess the urinary inflammatory, neurogenic, and oxidative stress biomarkers has achieved a satisfactory result [13,14,23]. Urinary biomarkers were assayed using commercially available microspheres with the Milliplex^®^ Human Cytokine/Chemokine Magnetic Bead-based panel kit. The levels of urine cytokines and chemokines such as MCP-1, eotaxin, TNF-α, and PGE2 were previously reported to be significantly higher in patients with IC/BPS than in the normal controls [29]. Most of the urinary biomarkers were significantly associated with MBC and the glomerulation grade in NHIC and the treatment outcome reported by the global response assessment. Among all biomarkers, TNF-α had the best sensitivity, specificity, positive predictive value, and negative predictive value.

Patients with HIC reportedly have significantly higher urinary levels of inflammatory proteins than those with NHIC [30]. Among these proteins, MCP-1, eotaxin, MIP-1β, TNF-α, and PGE2 were significantly different between the IC/BPS and control groups, while IL-8, CXCL10, BDNF, IL-6, and RANTES were significantly higher in the HIC group than in the NHIC group. An elevation in the urine levels of IL-8, CXCL 10, BDNF, IL-6, and RANTES in patients with IC/BPS should prompt physicians to consider the diagnosis of HIC [29]. The results of this study also showed that TNF-α ≥ CQ3, together with 8-OHdG ≥ CQ3 and 8-isoprostane ≥ CQ2, can be used as the screening biomarker to identify patients with IC/BPS and that adding CXCL 10 ≥ CQ2 or ≥ CQ3 can identify HIC from all patients with IC/BPS, showing satisfactory sensitivity and specificity results.

Aside from urine cytokines, the levels of urine oxidative stress biomarkers and inflammatory cytokine profiles are elevated in patients with IC/BPS, shown to be distinct from those of the controls and in each NHIC subgroup. Both 8-OHdG and 8-isoprostane showed a high diagnostic ability to distinguish ESSIC type 2 IC/BPS (NHIC) from the controls. In addition, they both showed positive and negative correlations with the glomerulation grade and MBC, respectively [14]. When we divided the NHIC subtypes according to the cystoscopic hydrodistention findings, patients with smaller MBC (<760 mL) had significantly higher levels of urinary inflammatory and oxidative stress biomarkers than those with larger MBC. The small MBC usually reflects a greater grade of inflammation in the IC/BPS bladders, indicating a less favorable overall treatment outcome in the long-term follow-up [29]. The increased levels of the urine oxidative stress biomarkers can be used to identify IC/BPS from the controls, but in differentiating HIC and NHIC, IP-10 has the best AUC among the other urinary biomarkers, suggesting that both HIC and NHIC had high oxidative stress status. Additionally, the HIC group had a higher inflammatory bladder condition than the NHIC group [30]. According to a recent study, the Epstein–Barr virus might play an important role in HIC pathophysiology [31]. Histopathological findings also revealed that NHIC with grade 3 glomerulation and HIC had higher inflammatory findings than those with grade 1 or 2 glomerulation [29]. In the present study, patients with small MBC and a higher glomerulation grade had a higher percentage of NHIC with IP-10 ≥ CQ3, further suggesting that the urinary level of inflammatory proteins such as CXCL10 can be used to assess the severity of inflammation in IC/BPS.

Furthermore, urinary biomarkers were significantly associated with glomerulation grade, MBC, VAS score, and bladder sensation. Most of the urinary biomarkers do not correlate with specific bladder histopathological findings; nevertheless, they are more important in the assessment of bladder condition than bladder histopathology [32]. Given that the inflammatory condition in IC/BPS varies widely, urinary biomarkers might have different levels of increase in HIC or NHIC. Using a single urinary biomarker for diagnosing IC/BPS, HIC, or NHIC with different bladder conditions is difficult. Therefore, using a cluster of urinary inflammatory and oxidative stress biomarkers might provide the most sensitive and specific diagnostic aid in identifying IC/BPS, HIC, or NHIC with different bladder conditions.

The current treatment of IC/BPS targets the specific pathophysiology of HIC or NHIC. Identifying HIC by severe bladder pain, very small functional capacity, and focal or diffuse bladder wall thickness enables the urologist to resect or fulgurate the Hunner’s lesion and thus quickly relieve the bladder pain symptoms [33]. When the bladder capacity is greatly reduced, augmentation enterocystoplasty is mandatory after partial cystectomy for HIC [34]. In the treatment of NHIC, hyaluronic acid is commonly used to supplement the defective urothelial barrier, although bladder inflammation cannot be reduced, bladder pain can be adequately relieved with less irritation by the urinary potassium [35]. Botulinum toxin injection, PRP injection, LESW therapy, or triamcinolone injection or low-dose prednisolone therapy are effective in reducing chronic inflammation and improve urothelial regeneration [11,36,37]. Urinary cytokine changes may serve as biomarkers to assess the treatment outcomes for IC/BPS subtypes such as high grade glomerulation, small MBC, or pelvic floor muscle pain without remarkable bladder pathology. If the cluster of urine biomarkers can identify the pathophysiology of IC/BPS, we may select appropriate bladder therapy for the right patient subtypes. A recent study analyzed the changes in urinary cytokines following various bladder therapies and explored their clinical significance in therapeutic mechanisms. PRP or BoNT-A exhibits anti-inflammatory effects, reflected by reductions in urinary cytokine levels, correlating with positive treatment outcomes. Urinary cytokine changes may play a role in evaluating the mechanisms of action of various treatments in patients with IC/BPS. In future research, we might find suitable patients with a high cluster of urinary biomarkers levels and provide appropriate bladder therapy to effectively improve their bladder conditions.

Recently, artificial intelligence and deep learning have been widely applied in disease prediction and the prognostic applications of IC/BPS [38,39]. The machine-learning decision tree model is reportedly more accurate in predicting the treatment outcome of patients with IC/BPS than logistic regression, and the 8-isoprostance, MCP-1, and 8-OHdG levels has the most important influence on accuracy [40]. According to logistic regression and multivariate analyses, the lower levels of urinary CXCL10, MCP-1, 8-OHdG, and 8-isoprostane were independent factors. In the machine-learning decision tree model, the combination of the urinary C-C motif chemokine 5, 8-isoprostane, TAC, MCP-1, and 8-OHdG could predict satisfactory results, with an accuracy value of 0.81. Urinary CXCL10, 8-OHdG, and 8-isoprostane were the independent factors that can predict satisfactory treatment results. These findings are consistent with the current study results wherein clustering TNF-α, 8-OHdG, and 8-isoprostane can identify IC/BPS and adding CXCL10 can distinguish HIC from NHIC.

LUTDs involve different levels of inflammatory responses, sensory hyperinnervation, and neurological and structural changes. These anatomical and functional changes will result in the release of different proteins into the urine. Therefore, the urinary biomarkers can be used to identify different LUTDs [13]. Using the urine chemokines CXCL-1 and IL-8, we can discriminate overactive bladder symptoms between DO and urinary tract infection in women. Urinary 8-OHdG and 8-isoprostane levels were significantly elevated in women with mixed DO than in those with genuine stress urinary incontinence. Urine TAC and prostaglandin E2 levels positively correlate with voiding detrusor pressure in patients with detrusor underactivity. Moreover, urinary BDNF and PGE2 levels were significantly higher in patients with DU with detrusor function recovery. Women with dysfunctional voiding had higher levels of urinary TNF-α and 8-OHdG. The urinary 8-isoprostane level increased in patients with idiopathic DO and neurogenic DO. In men, combining higher urinary levels of eotaxin and TNF-α can provide a satisfactory diagnostic value in discriminating IC/BPS from other LUTDs. Although current studies demonstrated that urinary biomarkers are significantly different between different LUTDs, using biomarkers to diagnose certain LUTDs or predict treatment outcomes still needs to be elucidated.

This study, for the first time, selected a cluster of urinary biomarkers such as TNF-α, 8-OHdG, and 8-isoprostane through machine learning to predict IC/BPS from the study cohort and added urinary IP-10 to identify HIC from the IC/BPS population. These urinary biomarkers were strongly associated with chronic inflammation in the HIC and NHIC. Using the urinary levels higher than the CQ3 or CQ3 of the controls, we found that a satisfactory percentage of patients can be screened out and treated as IC/BPS and that bladder therapy can be performed without the need to perform invasive diagnostic procedures. However, the accuracy of using a cluster of urinary biomarkers for different bladder conditions in NHIC remains uncertain, and the treatment outcome is still unachievable.

There are several limitations to this study. First, in this study, we selected 13 urinary biomarkers for the analysis of a predictive cluster of biomarkers for IC/BPS. The biomarkers were selected based on previously reported data, mainly on the inflammation and oxidative stress in IC/BPS. Other urinary biomarkers such as apoptosis pathway, adenosine triphosphate, nitric oxide, or metabolomics biomarkers are not included [41,42,43]. The results of the biomarker cluster might change if new biomarkers are added. Second, compared with the case number of IC/BPS, the non-IC/BPS patient number is relatively small. However, the entire dataset was randomly split into training and test sets and both models 1 and 2 were constructed using a multivariate logistic regression analysis with the training set and evaluated on the test set. These analytic process can decrease the potential bias to the minimum. Third, although the results of this study seem promising, we still need external validation to ensure its precision and clinical applicability in the future study. Further research is necessary to collect more patient data and adding other specific urinary biomarkers that can potentially predict satisfactory treatment results through different bladder therapies targeting urothelial dysfunction, neurogenic inflammation, or central sensitization.

## 5. Conclusions

Using a cluster of urinary biomarkers, namely TNF-α or 8-OHdG and 8-isoprostane, can identify patients with IC/BPS from a study cohort, and adding the urinary IP-10 can detect patients with HIC from an IC/BPS cohort. The accuracy rates were satisfactory in both the training and test sets. A higher level of urinary IP-10 also indicates a small MBC and higher glomerulation grade in patients with NHIC.

## Figures and Tables

**Table 1:** Comparison of the basic demographics between the training cohort and the test cohort.

	Total Enrollment	Training Set	Test Set	*p* Value ^a^
**Overall, N (%)**	422	297 (70%)	125 (30%)	-
Control group	46 (10.9%)	32 (10.8%)	14 (11.2%)	-
NHIC	334 (79.1%)	243 (81.8%)	91 (72.8%)	-
HIC	42 (9.95%)	22 (7.4%)	20 (16.0%)	-
**Clinical characteristics**				
Age (years)	53.9 ± 13.2	53.6 ± 13.0	54.6 ± 13.6	0.469
ICSI	12.4 ± 4.3	12.3 ± 4.3	12.6 ± 4.4	0.546
ICPI	11.7 ± 3.8	11.8 ± 3.7	11.5 ± 4.1	0.503
OSS	23.9 ± 7.9	23.8 ± 7.8	24.0 ± 8.0	0.837
VAS	5.3 ± 2.6	5.2 ± 2.6	5.3 ± 2.6	0.664
Glomerulation	1.8 ± 1.0	1.8 ± 0.9	1.8 ± 1.0	0.930
Qmax (mL/s)	12.6 ± 6.9	12.8 ± 7.2	12.1 ± 6.2	0.333
Volume (mL)	251.6 ± 134.7	254.3 ± 139.2	245.4 ± 123.9	0.537
PVR (mL)	28.9 ± 60.0	27.2 ± 58.7	33.1 ± 63.1	0.375
CBC (mL)	280.4 ± 129.4	281.9 ± 134.5	276.8 ± 117.0	0.698
MBC (mL)	686.7 ± 203.2	692.5 ± 202.2	672.9 ± 205.9	0.393

^a^ Comparison between both the training and validation groups. HIC: Hunner’s interstitial cystitis, NHIC: non-Hunner’s IC; ICSI: interstitial cystitis symptom index, ICPI: interstitial cystitis problem index, OSS: O’Leary–Sant score, VAS: visual analog score, Qmax: maximum flow rate, PVR: post-void residua, CBC cystometric bladder capacity, MBC: maximal bladder capacity under anesthesia.

**Table 2:** Comparison of urine cytokine concentrations between the training cohort and the test cohort.

	Total Enrollment	Training Set	Test Set	*p* Value
**Urine cytokines**				
Eotaxin	8.0 ± 10.3	7.5 ± 8.6	9.2 ± 13.6	0.206
IL-2	0.3 ± 0.2	0.3 ± 0.2	0.3 ± 0.2	0.154
IL-6	6.3 ± 22.8	5.8 ± 18.5	7.7 ± 30.7	0.433
IL-8	24.5 ± 47.3	22.3 ± 45.5	29.8 ± 51.2	0.139
CXCL-10 (IP-10)	33.0 ± 147.6	25.2 ± 123.5	51.4 ± 192.4	0.162
MCP-1	331.8 ± 497.4	333.6 ± 543.3	327.4 ± 367.7	0.907
MIP-1β	1.9 ± 4.9	1.6 ± 3.2	2.5 ± 7.58	0.196
RANTES	10.5 ± 47.6	7.3 ± 20.7	18.0 ± 81.2	0.150
TNFα	1.5 ± 1.7	1.3 ± 0.7	1.8 ± 2.9	0.056
NGF	0.2 ± 0.1	0.2 ± 0.1	0.2 ± 0.2	0.696
8-OHdG	58.7 ± 56.6	61.2 ± 60.0	52.7 ± 47.3	0.162
8-isoprostane	53.8 ± 100.0	51.5 ± 102.0	59.4 ± 95.4	0.455
TAC	992.9 ± 1167.4	977.3 ± 1125.4	1029.8 ± 1265.8	0.674

Abbreviations: IL: interleukin, CXCL10: C-X-C motif chemokine ligand 10, MCP-1: monocyte chemoattractant protein-1, MIP-1β: macrophage inflammatory proteins, RANTES: regulated on activation, normal T-cell expressed and secreted, TNF-α: tumor necrosis factor-alpha, NGF: nerve growth factor, 8-OHdG: 8-hydroxydeoxyguanosine. TAC: total antioxidant capacity, unit of biomarkers: pg/mL.

**Table 3:** Using an area under ROC (AUC) in the training set to identify urine cytokine predictors with better discriminatory power for model 1 (IC/BPS or not) and model 2 (HIC or NHIC).

	Identify IC/BPS		Identify HIC	
Urine Cytokines	AUC	95% CI	AUC	95% CI
Eotaxin	0.622	0.509–0.735	0.586	0.458–0.715
IL-2	0.009	0.001–0.017	0.512	0.385–0.639
IL-6	0.505	0.425–0.585	0.741	0.612–0.870
IL-8	0.573	0.465–0.682	0.736	0.614–0.857
IP-10	0.376	0.289–0.463	0.794	0.676–0.913
MCP-1	0.681	0.592–0.771	0.532	0.408–0.655
MIP1β	0.265	0.204–0.327	0.540	0.417–0.662
RANTES	0.402	0.323–0.481	0.658	0.530–0.786
TNF-α	0.775	0.696–0.855	0.535	0.411–0.659
NGF	0.210	0.116–0.304	0.475	0.334–0.615
8-OHdG	0.845	0.791–0.898	0.477	0.343–0.610
8-isoprostane	0.717	0.641–0.793	0.411	0.301–0.520
TAC	0.488	0.384–0.592	0.428	0.298–0.559

IC/BPS: Interstitial cystitis/bladder pain syndrome, HIC: Hunner’s interstitial cystitis, AUC: Area under the receiver operating characteristic curve, CI: Confidence interval. Other abbreviations: same as in Table 2, unit of biomarkers: pg/mL.

**Table 4:** Quartiles of urine cytokines concentration in the control group.

Urine Cytokines	CQ1	CQ2	CQ3
Eotaxin	1.60	3.41	6.59
IL-6	0.60	0.84	1.16
IL-8	1.31	4.42	12.63
IP-10	1.80	3.74	19.94
MCP-1	28.32	106.16	230.25
RANTES	1.97	3.80	8.04
TNF-α	0.62	0.73	0.95
8-OHDG	4.74	13.90	22.34
8-isoprostane	6.94	13.35	22.10

CQ1: Control group in the first quartile; CQ2: Control group in the second quartile; CQ3: Control group in the third quartile. Other abbreviations: same as in Table 2.

**Table 5:** Multivariable logistic regression for predicting IC/BPS (Model 1) and HIC (Model 2) using urinary cytokine concentrations.

Model 1-1: IC/BPS Prediction		
Urine cytokines	OR (95% CI)	*p*-value
Eotaxin ≥ CQ2 (3.41 pg/mL)	-	-
MCP-1 ≥ CQ2 (106.16 pg/mL)	-	-
TNF-α ≥ CQ2 (0.73 pg/mL)	4.421 (1.879–10.402)	0.001 **
8-OHDG ≥ CQ2 (13.9 pg/mL)	6.475 (2.790–15.024)	<0.001 **
8-isoprostane ≥ CQ2 (13.35 pg/mL)	2.709 (1.204–6.095)	0.016 **
**Model 1-2: IC/BPS prediction**		
Urine cytokines	OR (95% CI)	*p*-value
Eotaxin ≥ CQ3 (6.59 pg/mL)	-	-
MCP-1 ≥ CQ3 (230.25 pg/mL)	-	-
TNFα ≥ CQ3 (0.95 pg/mL)	24.038 (7.296–79.201)	<0.001 **
8-OHDG ≥ CQ3 (22.34 pg/mL)	29.077 (8.865–95.376)	<0.001 **
8-isoprostane ≥ CQ3 (22.1 pg/mL)	2.799 (1.015–7.718)	0.047 **
**Model 2-1: HIC prediction**		
Urine cytokines	OR (95% CI)	*p*-value
IL-6 ≥ CQ2 (0.84 pg/mL)	-	-
IL-8 ≥ CQ2 (4.42 pg/mL)	-	-
IP-10 ≥ CQ2 (3.74 pg/mL)	9.342 (3.060–28.522)	<0.001 **
RANTES ≥ CQ2 (3.80 pg/mL)	-	-
**Model 2-2: HIC prediction**		
Urine cytokines	OR (95% CI)	*p*-value
IL-6 ≥ CQ3 (1.16 pg/mL)	-	-
IL-8 ≥ CQ3 (12.63 pg/mL)	-	-
IP-10 ≥ CQ3 (19.94 pg/mL)	14.129 (5.350–37.316)	<0.001 **
RANTES ≥ CQ3 (8.04 pg/mL)	-	-

CQ2: Control group in the second quartile, CQ3: Control group in the third quartile, ** *p* < 0.05.

**Table 6:** Sensitivity and specificity of clustering urine cytokines in separate models for predicting IC and BPS.

	Training Set				Test Set			
Urine Cytokines	Sensitivity	Specificity	Accuracy	AUC	Sensitivity	Specificity	Accuracy	AUC
**Differential diagnosis of IC/BPS**								
TNF-α, 8-isoprostane, or 8-OHDG ≥ CQ2	100%	15.6%	90.9%	0.58	99.1%	14.3%	89.6%	0.57
TNF-α, 8-isoprostane, and 8-OHDG ≥ CQ2	53.6%	78.1%	56.2%	0.66	50.5%	78.6%	53.6%	0.65
TNF-α, 8-isoprostane, or 8-OHDG ≥ CQ3	100%	50.0%	94.6%	0.75	98.2%	35.7%	91.2%	0.67
TNF-α, 8-isoprostane, and 8-OHDG ≥ CQ3	35.5%	93.8%	41.8%	0.65	35.1%	92.9%	41.6%	0.64
TNF-α or 8-OHDG ≥ CQ3, or 8-isoprostane ≥ CQ2	100%	37.5%	93.3%	0.69	98.2%	21.4%	89.6%	0.60
TNF-α or 8-OHDG ≥ CQ3, and 8-isoprostane ≥ CQ2	76.2%	75.0%	76.1%	0.76	78.4%	71.4%	77.6%	0.75
TNF-α or 8-OHDG ≥CQ3, and 8-isoprostane ≥CQ3	62.3%	87.5%	65.0%	0.75	64.0%	78.6%	65.6%	0.71
**Differential diagnosis of HIC**								
IP-10 ≥ CQ2	81.8%	67.5%	65.7%	0.75	70.0%	67.0%	67.6%	0.69
IP-10 ≥ CQ3	68.2%	86.8%	85.3%	0.78	70.0%	80.2%	78.4%	0.75

CQ2: Control group in the second quartile, CQ3: Control group in the third quartile.

## Data Availability

The data of this study are available on request to the correspondence author.
